# Non-Authenticity of Spring Barley Genotypes Revealed in Gene Bank Accessions

**DOI:** 10.3390/plants11223059

**Published:** 2022-11-11

**Authors:** Antonín Dreiseitl, Marta Zavřelová

**Affiliations:** 1 Department of Integrated Plant Protection, Agrotest Fyto Ltd., 76701 Kroměříž, Czech Republic; 2 Gene Bank, Department of Plant Genetics and Breeding, Agricultural Research Institute Kroměříž Ltd., 76701 Kroměříž, Czech Republic

**Keywords:** *Hordeum vulgare*, gene banks, barley powdery mildew, *Blumeria graminis* f. sp. *hordei*, resistance gene postulation, mislabelled genetic resources, undesirable accession heterogeneity

## Abstract

Plant research and breeding depends on plant genotypes; therefore, genotype authenticity of accessions is the basic requirement for users of gene banks. Surprisingly, this extremely important topic is rarely reported in the scientific community. Non-authentic are accessions that are mislabelled and undesirable genotypes of heterogeneous accessions. In barley, we try to uncover both named problems on the basis of postulated major powdery mildew resistance genes. These are diverse, environmentally stable and their use is well documented and suitable for genotype characterization. In this contribution, we postulate resistance genes in 15 varieties represented by 157 derived lines of 32 accessions originating from seven foreign gene banks and compare these findings with previous results including those 15 identically labelled varieties from our domestic gene bank. We found that 37.5% of the gene bank accessions investigated herein were heterogeneous, and at least 20.0% were mislabelled. A large-scale molecular characterisation of varieties is now being carried out, and using authentic varieties must be one of the key requirements. Therefore, accessions of each variety from a minimum of three gene banks whose identity has been verified by reliable methods should be compared before starting new experiments. These will involve molecular varietal characterisation to serve as a foundation for future plant science research and effective crop improvement.

## 1. Introduction

Plants, especially crops and their genetic resources, are necessary for the survival and successful development of mankind. All research and breeding projects aiming at the selection of improved varieties or contribution to knowledge in plant sciences depend on plant genotypes. Their intraspecific genetic diversity represented by wild resources, landraces or bred varieties is usually preserved ex situ. Gene banks (GBs) implement standard procedures and staff strive to attain high levels of accuracy and reliability, but occasional human errors are inevitable [1]. The most serious mistakes result in heterogeneity of accessions due to contamination with other genotypes and non-authenticity of varieties (here also designated as mislabelled varieties) [2]. Both problems can increase mainly during reproduction of seed if not corrected.

We focus on GBs of major European self-pollinating cereals, i.e., spring barley (*Hordeum vulgare* L.) [3], including its wild subspecies *H. vulgare* subsp. *Spontaneum* (C. Koch) Tell. [4], winter barley [5,6] and wheat (*Triticum aestivum* L.) [7]. It was surprising that although their samples rank among the most numerous in gene banks, we found no references dealing with non-authenticity of varieties and undesirable non-original heterogeneity of these cereal accessions. Nevertheless, occasional results from GBs of other plant species showed the problem is common and of concern.

Af Satra et al. [8] genotyped apples grown in different locations and confirmed the identity of multiple samples with the same cultivar name, but also identified several mislabelled samples. Similarly, there were possible mislabelled accessions in the world collections of wild *Cicer* (chick-pea) germplasm by Shan et al. [9]. Jreisat and Laten [10] reported that 4 of the 13 specimens of clover (*Trifolium*) appear to be mislabelled or misidentified. Zhang et al. [11] applied molecular methods to determine species’ identity in rice (*Oryza*) and revealed that 17% of the 53 seed accessions from GBs or field collections were mislabelled. In a GB of yam (*Discorea*), whose tubers are used as a staple food or natural medicine, 20.6% of the total 3156 GB accessions were not true to type and mislabelling probably played an important part [12]. Van de Wouw et al. [13] found high non-authenticity of lettuce (*Lactuca sativa*) cultivars in gene bank collections; this was especially true for the oldest varieties, but even for cultivars released from the 1960s to 1990 it was estimated that approximately 10% were not authentic. In the germplasm collection of ornamental crop *Hydrangea macrophylla,* the authors deduced that 36% of the tested plants were mislabelled [14].

Breeding barley resistant to powdery mildew caused by an ascomycete fungus *Blumeria graminis* f. sp. *hordei* (*Bgh*) has been based on major genes [15] and their exploitation and utilization was closely monitored and summarized [16,17,18]. While most of the barley traits exhibit significant genotype × environment interactions [19], major resistance genes are stable, and among classical methods, knowledge of their presence in varieties is suitable for genotype characterisation.

Based on previous research [2] and due to the natural exchange of curators, a revision of domestic spring and winter barley GBs is being carried out focused mainly on assessing homogeneity and authenticity of the stored accessions. We postulate powdery mildew resistance genes in the accessions and compare these with earlier findings of these genes and with the year of their first use in commercial varieties [16,17]. First, we performed resistance tests of all accessions of domestic spring and winter barley core collections (CCs) and discovered that most of them were heterogeneous [3,5]. Based on these tests, the resistance genes in homogeneous and some heterogeneous accessions could be postulated. Subsequently, five single-seed progenies (SSPs) grown from seed of heterogeneous accessions sourced from both GBs were tested and resistance genes of individual lines were identified [3,6]. The aim of this follow-up work was to investigate accessions in other international GBs.

## 2. Results

In total, 157 spring barley SSPs (lines) derived from 15 selected varieties originating from seven foreign GBs were tested with 53 isolates of the pathogen, which resulted in 24 infection response arrays (IRAs) reflecting phenotypes of 14 known *Ml* genes, of them 11 single, 12 gene combinations and an IRA confirming the absence of any major resistance gene. Eight isolates were sufficient to characterise the IRAs (Table 1). In two accessions, unknown (u) resistances were found. Postulated resistance genes in accessions originating from different GBs were compared mutually and also with previous results of 75 SSPs tested from the same 15 varieties maintained in the domestic GB (Table 2). Thus, resistances of 232 genotypes were analysed and presented in Table S1. From 32 accessions lodged in foreign GBs, 12 (37.5%) were heterogeneous and contained two or more genotypes, and at least six out of 30 accessions (except two accessions of Schwarzenberg Gerste 21—see Specific Discussion), i.e., 20.0% were mislabelled. Moreover, in three (10.0%) heterogeneous accessions (Diamant—SVK, Rupee—GBR and Trumf—USA), non-authentic genotypes predominated and, therefore, they can also be considered as mislabelled. Detailed information about resistance genes found in individual varieties, their accessions originating from different GBs and derived SSPs are presented in Specific Discussion.

## 3. Discussion

### 3.1. Specific Discussion

**Abyssinian 1102** (in GBs designated also HOR2551, HOR3036, L94 or BBA1465) is an Ethiopian landrace well known as the source of *mlo11*, the most important recessive resistance gene against barley powdery mildew and first commercially used in Atem—a variety released in the Netherlands in 1979 [20]. However, none of the three genotypes found previously [3] carried *mlo* and, besides some others, this accession inspired our present research. In the current study, we found *mlo* in both homogeneous accessions from GBR and USA.

According to Brown and Jørgensen [16], **Asse** contains *Mlg*. We found *Mla8* in the USA accession and *Mlra*—a typical gene of winter barleys accompanied here with an unknown resistance—in our domestic GB [3]. Asse is a German variety of more than sixty years old, and because no correct genotype was found in accessions from both GBs, we will request a sample from a German GB to replace our own accession if its true identity is confirmed.

**Diamant** was one of most important domestic varieties released in 1965 because its semi-dwarf sdw1.d allele [21] has made a positive contribution to the breeding of spring barley worldwide. In a homogeneous GBR accession *Mla8, MlHe2* were established, but in the domestic GB, four genotypes were recognised [3]. Two lines were certainly not genuine Diamant since they contained *mlo* and *Mla7, MlLa*, which were combined in barley cultivars later [16]. One SSP carried *Mla8* and another *Mla8, MlHe2*. In the accession from SVK, there were three genotypes; two could be excluded for the same reason, and in the third, a combination of genes *Mla8, MlHe2* was postulated. Dreiseitl and Jørgensen [17] did not record any *Ml* gene in Diamant, but at that time, no *Bgh* isolates detecting *Mla8* nor *MlHe2* were available for the given tests. Diamant was created by mutation from Valticky carrying *Mla8, MlHe2* [3], and based on these results one SSP from each CZE and SVK as well as a GBR accession can be accepted as Diamant.

In accessions of **Donaria Ackermanns** (sometimes known as Ackermanns Donaria or Donaria) from three GBs (DEU, GBR and USA), the same gene combination as in Diamant from GBR was found (*Mla8, MlHe2*). This combination should be correct, although in the domestic GB accession, *MlCh, MlHe2* were present [3] because Jørgensen and Jensen [22] found *Mla8* in this variety (Donaria).

**Emir** is a well-known standard variety carrying *Mla12* [23] and this gene was present in all three accessions from GBs DEU, GBR and USA. In lines derived from the domestic GB accession, three SSPs carried *Mla8,* and two were without any powdery mildew resistance gene (denoted ‘*none*’). It indicates that the domestic accession was heterogeneous and contained only non-authentic genotypes.

Besides previous tests of the CZE **Gerda** [3], accessions of this variety from three foreign GBs were tested. In nine out of ten SSPs from GBR and USA, *Mla6, Mlg* were found, and this gene combination corresponds with results compiled in the European catalogue [16]. In SSPs derived from heterogeneous accessions of our domestic and Polish GBs, four different genotypes were detected, but all differed from those published earlier [16].

Different results exist for Hana and Hanna. The names are derived from the name of a fertile Moravian region of the Czech Republic (Haná) and had been assigned to varieties of different crops including several high-quality malting barleys. Heils Hanna was one of the first varieties where *Mla8* was recorded [24], and it was recorded as the main donor of this gene [23]. In our previous study, we found *Mla8, MlHe2* in four **Hana** genotypes [3]—an identical gene combination as in Diamant, one of two parents of this variety—and in one SSP, *Mlg, MlHe2* were postulated. This gene combination was also found in four SSPs from USA, where, in contrast, one SSP carrying *Mla8*, *MlHe2* occurred. Dreiseitl and Jørgensen [17] did not find any *Ml* gene in Hana, but as in the case of Diamant, no *Bgh* isolates for detecting *Mla8* nor *MlHe2* were available for these tests. Hence, *Mla8*, *MlHe2* could be the actual genotype of Hana, but the question of why *Mlg, MlHe2* was detected in both GBs remains.

The resistance gene *Mlh* was named after **Hanna**, but in this test, *Mlg* was present. Both these results had already been confirmed several times [25] for identically named but surely different varieties. *Mlg* in Hanna was discovered in our previous study [3] and here, too, in the DEU accession, whereas in all SSPs of the Hungarian accession and in one SSP from POL, *MlCh, MlHe2* were postulated; in four other Polish SSPs *Mla8* was present. It is likely, therefore, that our domestic accession can be accepted as one of Hanna varieties.

**Rupee** is an important donor of the resistance gene *Mla13* [23]. However, in our previous tests [3], only an unknown resistance different from *Mla13* was found. In the current research, a heterogeneous accession from the DEU GB, four SSPs possessed *Mla13*, whereas in the GBR accession only two SSPs had the same gene as all SSPs from USA.

Our previous [3] and two present accessions of **Schwarzenberg Gerste 21** tested here were homogeneous, but each of them carried a different resistance (including *none* in DEU). Because this variety is old, we can exclude the authenticity of the domestic (CZE) accession carrying *Mla6, Mlg*. Conversely, both ‘*none*’ and *Mla8* are typical for varieties released before the advent of powdery mildew breeding. Therefore, we cannot establish which of these two accessions could be the genuine variety.

In five lines of **Trumpf** investigated previously, three different genotypes were found [3] but none of them was identical to the resistance of the same variety designated Triumph [16] nor with an identical resistance of Trumpf from a working collection of the first author. In the present test, there was a heterogeneous accession from USA and two homogeneous accessions from German and Polish GBs with different resistance genes. The accession from DEU and two SSPs from USA have the same main gene published by Brown and Jørgensen [16] for this variety. Trumpf from the Polish GB has *Mla8*, frequently present in old varieties including the old Czech variety Triumf [3].

In our previous tests [3], **Vega Abed** was homogeneous and carried *Mla13,* whereas its resistance is purported to be *MlLa* [16]. Testing of 15 SSPs from three foreign GBs (DEU, POL and SWE) revealed *Mla8* in 10, *Mla8, MlLa* in two and *Mla8, MlHe2, MlLa* in three of them. Hence, the *MlLa* was present in all three GB accessions but in only a minority of SSPs. It is probable that this variety originally included all three lines found here.

Three varieties (**Black Hull-less, Falcon** and **Manchuria**) have identical resistances (Manchuria ‘*none*’) as in the domestic GBs [3]. Because we have no other information about the resistance of these varieties, it is possible that all these accessions, including our original domestic one, were correct.

### 3.2. General Discussion

Diez et al. [26] summarized several problems of GBs such as the existence of too many of them, detected and undetected varietal duplication, infrequent seed multiplication, insufficient phenotyping and genotyping. However, the authors did not mention mislabelling or heterogeneity of accession. Despite this omission, genotype authenticity of accessions certainly is a fundamental pre-requisite for users.

Dreiseitl [4] tested powdery mildew resistance in GB accessions of the progenitor of cultivated barley and 40.0% of them were heterogeneous. Czembor [27] studied 41 landraces collected in Morocco, and because of their heterogeneity, he used the designation “landrace populations”. In 1996–2022, 146 spring barley varieties were newly registered in the Czech Republic, and according to their powdery mildew resistance genes 10 (6.8%) comprised two or more lines. Such heterogeneity of wild relative collections, landraces and multiline varieties can be considered as primary, whereas the presence of different genotypes due to mechanical genotype contamination or cross pollination represents secondary and undesirable heterogeneity.

Accession heterogeneity ranges from a very low proportion of an admixture up to 100% of many non-authentic genotypes. Previous tests of 223 accessions of the Czech GB CC of spring barley revealed 133 (59.6%) heterogeneous accessions [3] and in the Czech GB CC of winter barley out of 172 accessions 147 (85.5%) were heterogeneous [5]. This might even be an underestimate, since heterogeneity could not be verified in all accessions because groups of varieties with an identical resistance were present in both CCs and their mutual contamination within these groups could not be detected. Furthermore, it was difficult to identify those genotypes that occurred at a low frequency because testing of “only” five lines was insufficient to fully characterise the heterogeneity. Based on the present results of 32 accessions from foreign gene banks, 12 (37.5%) were heterogeneous.

Accurate genotyping is essential for research and plant breeding, so it is surprising that minimal attention is paid to this fundamental topic in the scientific literature, perhaps because non-authentic genotypes are rarely detected in time. However, for that reason, the solution of many research projects must result in improper findings, conclusions and recommendations. In our study, at least 6 out of 30 (20.0%) foreign spring barley accessions (apart from two accessions of Schwarzenberg Gerste 21) were not authentic.

In GBs, novel procedures and services should be used, including selection of genetically purified lines, creation of gene cassettes from originally heterogeneous varieties and verification of genotype authenticity and seed purity of accessions using suitable and environmentally stable methods after each reproduction cycle. Duplication of accessions is also an important problem for GBs [28], but during the establishment of genotype identity and their relevant characteristics the existence of duplicates may be necessary.

Authenticity of varieties is important for their large-scale molecular characterisation, which should serve as a new dataset for subsequent experiments and development of biological research [29,30]. Individual plants from pre-selected and morphologically identical SSPs are often used for this purpose. However, our results show that for all, but mainly for older varieties, a detailed investigation to ensure their true and homogeneous genotypes is needed. Therefore, accessions originating from a minimum of three different GBs and whose identity is verified by reliable methods should be compared before performing experiments focused on new varietal characterisations, especially those involving molecular analyses. It is surely time-consuming, but success of all research and breeding projects depends on well-characterised authentic plant genotypes.

## 4. Materials and Methods

From foreign GBs, we ordered accessions of those varieties which showed a discrepancy among results reported from different sources [3]. Then, we first performed resistance tests in obtained accessions; second, we postulated their resistance genes; third, we compared results of identically labelled accessions from different GBs, including domestic ones, and fourth, we compared results with the published data.

### 4.1. Plant Material

Fifteen varieties of spring barley were tested, each obtained from one to three out of seven cooperating foreign GBs, totaling 32 accessions (Table 1). From seed of each accession, five SSPs were harvested, apart from Black Hull-less, from which only two SSPs were available. In total, 157 SSPs (lines) were tested.

### 4.2. Pathogen Isolates

For resistance tests, 53 selected reference isolates of *Bgh* were used, which had been collected in 11 countries over a period of 64 years (1953–2016) and comprised the global virulence/avirulence diversity of the pathogen. The responses of 35 standard barley genotypes carrying different specific resistance genes to these isolates have been described earlier [31]. Before inoculation, all isolates were checked for their purity, and their correct pathogenicity phenotypes were verified on standard barley lines [32]. The isolates were multiplied on leaf segments of the susceptible cv. Stirling.

### 4.3. Testing Procedure

Approximately 50 seeds of each SSP were sown in two pots (80 mm diameter) filled with a gardening peat substrate and placed in a mildew-proof greenhouse under natural daylight. The primary leaves were excised when the second leaves were emerging, and leaf segments of about 15 mm long were cut from the middle part of healthy fully expanded leaves. Three segments of each accession were placed on the surface of the media (0.8% water agar containing 40 mg−L of benzimidazole—a leaf senescence inhibitor) in a 150 mm Petri dish. Leaf segments were placed adjacent to each other along with four segments of Stirling oriented diagonally with their adaxial surfaces facing upward.

For inoculation, a cylindrical metal settling tower of 150 mm diameter and 415 mm in height closed at the top was used, and a dish with segments was placed at the bottom of the tower. Conidia of each isolate taken from a leaf segment of the susceptible variety with fully developed pathogen colonies were shaken onto a square piece 40 × 40 mm of black paper to visually control the amount of inoculum deposited. Then, the paper was rolled to form a blowpipe, and conidia of the isolate were blown through a side hole of 13 mm diameter with its centre 50 mm from the upper end into the settling tower over the Petri dish at a concentration of ca. 10 conidia mm^−2^. The dishes with inoculated leaf segments were incubated at 20 ± 1 °C under cool-white fluorescent lamps providing 12 h light at 30 ± 5 μmol m^−2^ s^−1^.

### 4.4. Evaluation

**Figure 1:**
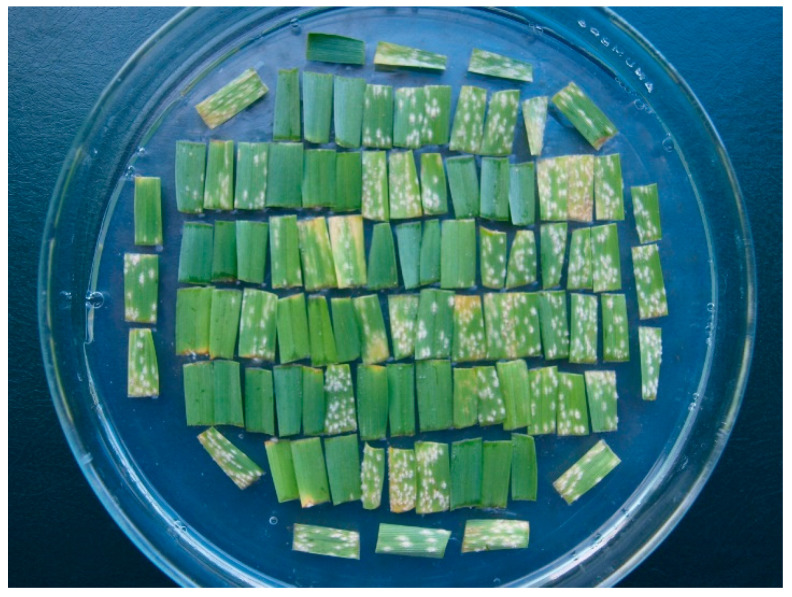
Petri dish with triplets of leaf segments of 30 barley varieties and four diagonally placed segments of a check susceptible variety viewed seven days after inoculation with a powdery mildew isolate.

Seven days after inoculation, infection responses (IR = phenotype of SSP x isolate interaction) were scored on a scale of 0–4 [33], where 0 = no mycelium and sporulation, and 4 = strong mycelial growth and sporulation (Figure 1). IRs 3, 3–4 and 4 were considered susceptible. Based on the gene-for-gene model [34], the resistance genes in SSPs were postulated by comparing their IRAs with earlier determined IRAs of standard barley genotypes possessing known resistance genes. During phenotyping, special attention was paid to IRs 2–3 and 3 which pose the greatest risk of error in distinguishing between resistance and susceptibility [35]. Other details of Materials and Methods have been recently described [36].

## 5. Conclusions

- Accurate genotyping is essential for research and plant breeding, so it is surprising that minimal attention is paid to this fundamental topic in the scientific literature.
- Breeding barley resistant to powdery mildew has been based on major genes and their exploitation and utilization was monitored.
- While most of the barley traits exhibit significant genotype × environment interactions, major resistance genes are stable and knowledge of their presence in varieties is suitable for genotype characterisation.
- In total, 157 spring barley lines derived from 15 varieties which showed a discrepancy among previous results reported from different sources and originating from seven foreign GBs were tested here with 53 isolates of the pathogen.
- Fourteen known *Ml* genes, 12 of their combinations and two unknown genes were found.
- We compared our results of identically labelled accessions from different GBs, including domestic ones with published data.
- From 32 accessions lodged in foreign GBs, 12 (37.5%) were heterogeneous containing two or more genotypes, and at least 6 out of 30 accessions, i.e., 20.0% were mislabelled.
- Our results show that a detailed investigation to ensure true and homogeneous variety genotypes is needed; due to non-authentic genotypes, the solution of many research projects must result in improper findings, conclusions and recommendations.
- Authentic identity of varieties should be verified by reliable methods before performing experiments focused on new varietal characterisations, especially those involving molecular analyses.

## Figures and Tables

**Table 1:** Infection response arrays (IRAs) produced by eight *Blumeria graminis* f. sp. *hordei* (*Bgh*) isolates on 157 barley genotypes and the corresponding powdery mildew resistance genes.

*Ml* Gene(s)	*Bgh* Isolates, Their Country of Origin and Year of Collection							
	Race I	J-462	EA30	U-54	I-20	M-3	GH	X-30
	JAP ^1^	ISR	SWE	URQ	CZE	CZE	AUS	CZE
	1953	1979	1976	2005	2011	2014	2005	2012
None	4	4	4	4	4	4	4	4
*a6*	0	4	4	0	4	4	0	4
*a6, g*	0	4	0	0	0	4	0	4
*a6, La*	0	4	0	0	0	4	0	2–3
*a7*	0	0	1	0	4	4	0	4
*a7, g*	0	0	1	0	0	4	0	4
*a7, k1, g*	0	0	1	0	0	2	0	2
*a7, k1, La*	0	0	1	0	0	4	0	2–3
*a7, La*	0	0	1	0	4	4	0	2–3
*a8*	0	4	4	4	4	4	4	4
*a8, He2*	0	4	4	2–3	4	4	4	4
*a8, He2, La*	0	4	2–3	2–3	4	4	2–3	2–3
*a8, La*	0	4	2–3	4	4	4	2–3	2–3
*a9*	0	4	0	0	0	0	0	0
*a12*	1	4	4	0	4	4	1	4
*a13*	0	0	0	0	4	4	0	4
*a13, g*	0	0	0	0	0	4	0	4
*Ch*	2	4	4	4	4	4	4	4
*Ch, He2*	2	4	4	2–3	4	4	4	4
*g*	0	4	0	4	0	4	4	4
*g, He2*	0	4	0	2–3	0	4	4	4
*mlo*	0(3) ^2^	0(3)	0(3)	0(3)	0(3)	0(3)	0(3)	0(3)
*n*	4	4	1–2	4	1–2	1–2	1–2	1–2
*ra*	4	4	0	4	4	4	4	4

^1^ Country of isolate origin: AUS—Australia, CZE—Czech Republic, ISR—Israel, JAP—Japan, SWE—Sweden, URQ—Uruguay. ^2^ Wild phenotype, rare colonies are denoted in parentheses.

**Table 2:** Thirty-two accessions representing 15 spring barley varieties obtained from seven foreign gene banks compared with previously tested accessions from a domestic gene bank.

Variety	CZE ^1,2^	USA	DEU	GBR	POL	HUN	SVK	SWE	Sum
Abyssinian 1102	T ^b^	T ^a^		T ^a^					2
Asse	T ^c^	T ^b^							1
Black Hull-less	T ^a^	T ^a^							1
Diamant	T ^b^			T ^a^			T ^b^		2
Donaria Ackermans	T ^b^	T ^a^	T ^a^	T ^a^					3
Emir	T ^b^	T ^a^	T ^a^	T ^a^					3
Falcon	T ^a^		T ^a^						1
Gerda	T ^b^	T ^a^		T ^a^	T ^c^				3
Hana	T ^a^	T ^b^							1
Hanna	T ^a^		T ^a^		T ^b^	T ^c^			3
Manchuria	T ^a^	T ^a^							1
Rupee	T ^b^	T ^a^	T ^a^	T ^c^					3
Schwarzenb. Gerste 21	T ^b^		T		T				2
Trumpf	T ^d^	T ^b^	T ^a^		T ^c^				3
Vega Abed	T ^b^		T ^a^		T ^a^			T ^a^	3
Sum	**15**	**10**	**8**	**6**	**5**	**1**	**1**	**1**	**32**

^1^ Previous results [3]. ^2^ Country of gene bank: CZE—Czech Republic, USA—United States, DEU—Germany, GBR—United Kingdom, POL—Poland, HUN—Hungary, SVK—Slovakia, SWE—Sweden. T = five single-seed progenies were tested from each accession except Black Hull-less where only two progenies were available; highlighted accessions were heterogeneous. ^a–d^ Postulated different powdery mildew resistance genotypes where genotype a is correct.

## Data Availability

Not applicable.

## Supplementary Materials

The following supporting information can be downloaded at: https://www.mdpi.com/article/10.3390/plants11223059/s1, Table S1. Accessions of 15 spring barley varieties originating from seven cooperating gene banks, 157 derived single-seed progenies (SSPs) and their postulated *Ml* powdery mildew resistance genes, and their comparison with 75 SSPs of the same varieties from domestic gene bank studied previously.
