# Supplementary material for: Non-Authenticity of Spring Barley Genotypes Revealed in Gene Bank Accessions
Source: Plants (Basel). 2022 Nov 11;11(22):3059. doi: 10.3390/plants11223059 (PMC9698254; doi:10.3390/plants11223059)
Supplement: Supplementary file 1 [file plants-11-03059-s001.zip › plants-2004492-supplementary.pdf]

plants

MDPI

Table S1. Accessions of 15 spring barley varieties originating from seven cooperating gene banks, 157 derived single seed progenies (SSPs) and their postulated *Ml* powdery mildew resistance genes, and their comparison with 75 SSPs of the same varieties from domestic gene bank studied previously.

Accession	SSP	Gene Bank	*Ml* Resistance Gene(s)
Abyssinian 1102[1]	1	*CZE*[2]	*none*
Abyssinian 1102	2	*CZE*	*a8, He2*
Abyssinian 1102	3	*CZE*	*a8, He2*
Abyssinian 1102	4	*CZE*	*a7, g*
Abyssinian 1102	5	*CZE*	*a8, He2*
Abyssinian 1102	1	GBR	*mlo*
Abyssinian 1102	2	GBR	*mlo*
Abyssinian 1102	3	GBR	*mlo*
Abyssinian 1102	4	GBR	*mlo*
Abyssinian 1102	5	GBR	*mlo*
Abyssinian 1102 (L94)	1	USA	*mlo*
Abyssinian 1102 (L94)	2	USA	*mlo*
Abyssinian 1102 (L94)	3	USA	*mlo*
Abyssinian 1102 (L94)	4	USA	*mlo*
Abyssinian 1102 (L94)	5	USA	*mlo*
Asse	1	*CZE*	*ra, u*
Asse	2	*CZE*	*ra, u*
Asse	3	*CZE*	*ra, u*
Asse	4	*CZE*	*ra, u*
Asse	5	*CZE*	*ra, u*
Asse	1	USA	*a8*
Asse	2	USA	*a8*
Asse	3	USA	*a8*
Asse	4	USA	*a8*
Asse	5	USA	*a8*
Black Hull-less	1	*CZE*	*n*
Black Hull-less	2	*CZE*	*n*
Black Hull-less	3	*CZE*	*n*
Black Hull-less	4	*CZE*	*n*
Black Hull-less	5	*CZE*	*n*
Black Hull-less	1	USA	*n*
Black Hull-less	2	USA	*n*
Diamant	1	*CZE*	*mlo*
Diamant	2	*CZE*	*mlo*
Diamant	3	*CZE*	*a8*
Diamant	4	*CZE*	*a7, La*
Diamant	5	*CZE*	*a8, He2*

Diamant	1	GBR	*a8, He2*
Diamant	2	GBR	*a8, He2*
Diamant	3	GBR	*a8, He2*
Diamant	4	GBR	*a8, He2*
Diamant	5	GBR	*a8, He2*
Diamant	1	SVK	*mlo*
Diamant	2	SVK	*mlo*
Diamant	3	SVK	*a6, La*
Diamant	4	SVK	*a8, He2*
Diamant	5	SVK	*mlo*
Donaria Ackermanns	1	*CZE*	*Ch, He2*
Donaria Ackermanns	2	*CZE*	*Ch, He2*
Donaria Ackermanns	3	*CZE*	*Ch, He2*
Donaria Ackermanns	4	*CZE*	*Ch, He2*
Donaria Ackermanns	5	*CZE*	*Ch, He2*
Donaria Ackermanns	1	DEU	*a8, He2*
Donaria Ackermanns	2	DEU	*a8, He2*
Donaria Ackermanns	3	DEU	*a8, He2*
Donaria Ackermanns	4	DEU	*a8, He2*
Donaria Ackermanns	5	DEU	*a8, He2*
Donaria Ackermanns	1	GBR	*a8, u*
Donaria Ackermanns	2	GBR	*a8, He2*
Donaria Ackermanns	3	GBR	*a8, He2*
Donaria Ackermanns	4	GBR	*a8, He2*
Donaria Ackermanns	5	GBR	*a8, He2*
Donaria Ackermanns	1	USA	*a8, He2*
Donaria Ackermanns	2	USA	*a8, He2*
Donaria Ackermanns	3	USA	*a8, He2*
Donaria Ackermanns	4	USA	*a8, He2*
Donaria Ackermanns	5	USA	*a8, He2*
Emir	1	*CZE*	*none*
Emir	2	*CZE*	*none*
Emir	3	*CZE*	*a8*
Emir	4	*CZE*	*a8*
Emir	5	*CZE*	*a8*
Emir	1	DEU	*a12*
Emir	2	DEU	*a12*
Emir	3	DEU	*a12*
Emir	4	DEU	*a12*
Emir	5	DEU	*a12*
Emir	1	GBR	*a12*
Emir	2	GBR	*a12*

Emir	3	GBR	*a12*
Emir	4	GBR	*a12*
Emir	5	GBR	*a12*
Emir	1	USA	*a12*
Emir	2	USA	*a12*
Emir	3	USA	*a12*
Emir	4	USA	*a12*
Emir	5	USA	*a12*
Falcon	1	*CZE*	*Ch*
Falcon	2	*CZE*	*Ch*
Falcon	3	*CZE*	*Ch*
Falcon	4	*CZE*	*Ch*
Falcon	5	*CZE*	*Ch*
Falcon	1	DEU	*Ch*
Falcon	2	DEU	*Ch*
Falcon	3	DEU	*Ch*
Falcon	4	DEU	*Ch*
Falcon	5	DEU	*Ch*
Gerda	1	*CZE*	*g*
Gerda	2	*CZE*	*a8*
Gerda	3	*CZE*	*a7, k1, g*
Gerda	4	*CZE*	*a7, k1, g*
Gerda	5	*CZE*	*a7, k1, g*
Gerda	1	GBR	*a6, g*
Gerda	2	GBR	*a6, g*
Gerda	3	GBR	*a6*
Gerda	4	GBR	*a6, g*
Gerda	5	GBR	*a6, g*
Gerda	1	POL	*a8, He2*
Gerda	2	POL	*a8, He2*
Gerda	3	POL	*a8*
Gerda	4	POL	*a8, He2*
Gerda	5	POL	*a8, He2*
Gerda	1	USA	*a6, g*
Gerda	2	USA	*a6, g*
Gerda	3	USA	*a6, g*
Gerda	4	USA	*a6, g*
Gerda	5	USA	*a6, g*
Hana	1	*CZE*	*a8, He2*
Hana	2	*CZE*	*a8, He2*
Hana	3	*CZE*	*a8, He2*
Hana	4	*CZE*	*a8, He2*

Hana	5	*CZE*	*g, He2*
Hana	1	USA	*g, He2*
Hana	2	USA	*g, He2*
Hana	3	USA	*a8, He2*
Hana	4	USA	*g, He2*
Hana	5	USA	*g, He2*
Hanna	1	*CZE*	*g*
Hanna	2	*CZE*	*g*
Hanna	3	*CZE*	*g*
Hanna	4	*CZE*	*g*
Hanna	5	*CZE*	*g*
Hanna	1	DEU	*g*
Hanna	2	DEU	*g*
Hanna	3	DEU	*g*
Hanna	4	DEU	*g*
Hanna	5	DEU	*g*
Hanna	1	HUN	*Ch, He2*
Hanna	2	HUN	*Ch, He2*
Hanna	3	HUN	*Ch, He2*
Hanna	4	HUN	*Ch, He2*
Hanna	5	HUN	*Ch, He2*
Hanna	1	POL	*Ch, He2*
Hanna	2	POL	*a8*
Hanna	3	POL	*a8*
Hanna	4	POL	*a8*
Hanna	5	POL	*a8*
Manchuria	1	*CZE*	*none*
Manchuria	2	*CZE*	*none*
Manchuria	3	*CZE*	*none*
Manchuria	4	*CZE*	*none*
Manchuria	5	*CZE*	*none*
Manchuria	1	USA	*none*
Manchuria	2	USA	*none*
Manchuria	3	USA	*none*
Manchuria	4	USA	*none*
Manchuria	5	USA	*none*
Rupee	1	*CZE*	*u*
Rupee	2	*CZE*	*u*
Rupee	3	*CZE*	*u*
Rupee	4	*CZE*	*u*
Rupee	5	*CZE*	*u*
Rupee	1	DEU	*a8*

Rupee	2	DEU	*a13*
Rupee	3	DEU	*a13*
Rupee	4	DEU	*a13*
Rupee	5	DEU	*a13*
Rupee	1	GBR	*Ch*
Rupee	2	GBR	*Ch*
Rupee	3	GBR	*a13*
Rupee	4	GBR	*a13*
Rupee	5	GBR	*a8*
Rupee	1	USA	*a13*
Rupee	2	USA	*a13*
Rupee	3	USA	*a13*
Rupee	4	USA	*a13*
Rupee	5	USA	*a13*
Schwarzenberg Gerste 21	1	*CZE*	*a6, g*
Schwarzenberg Gerste 21	2	*CZE*	*a6, g*
Schwarzenberg Gerste 21	3	*CZE*	*a6, g*
Schwarzenberg Gerste 21	4	*CZE*	*a6, g*
Schwarzenberg Gerste 21	5	*CZE*	*a6, g*
Schwarzenberg Gerste 21	1	DEU	*none*
Schwarzenberg Gerste 21	2	DEU	*none*
Schwarzenberg Gerste 21	3	DEU	*none*
Schwarzenberg Gerste 21	4	DEU	*none*
Schwarzenberg Gerste 21	5	DEU	*none*
Schwarzenberg Gerste 21	1	POL	*a8*
Schwarzenberg Gerste 21	2	POL	*a8*
Schwarzenberg Gerste 21	3	POL	*a8*
Schwarzenberg Gerste 21	4	POL	*a8*
Schwarzenberg Gerste 21	5	POL	*a8*
Trumpf	1	*CZE*	*a9*
Trumpf	2	*CZE*	*a13, g*
Trumpf	3	*CZE*	*a13, g*
Trumpf	4	*CZE*	*a9*
Trumpf	5	*CZE*	*a7, k1, La*
Trumpf	1	DEU	*a7*
Trumpf	2	DEU	*a7*
Trumpf	3	DEU	*a7*
Trumpf	4	DEU	*a7*
Trumpf	5	DEU	*a7*
Trumpf	1	POL	*a8*
Trumpf	2	POL	*a8*
Trumpf	3	POL	*a8*

Trumpf	4	POL	*a8*
Trumpf	5	POL	*a8*
Trumpf	1	USA	*a7*
Trumpf	2	USA	*a7*
Trumpf	3	USA	*a8, He2*
Trumpf	4	USA	*a8, He2*
Trumpf	5	USA	*a8, He2*
Vega Abed	1	*CZE*	*a13*
Vega Abed	2	*CZE*	*a13*
Vega Abed	3	*CZE*	*a13*
Vega Abed	4	*CZE*	*a13*
Vega Abed	5	*CZE*	*a13*
Vega Abed	1	DEU	*a8, He2, La*
Vega Abed	2	DEU	*a8*
Vega Abed	3	DEU	*a8*
Vega Abed	4	DEU	*a8*
Vega Abed	5	DEU	*a8*
Vega Abed	1	POL	*a8*
Vega Abed	2	POL	*a8*
Vega Abed	3	POL	*a8*
Vega Abed	4	POL	*a8, La*
Vega Abed	5	POL	*a8, He2, La*
Vega Abed	1	SWE	*a8*
Vega Abed	2	SWE	*a8, He2, La*
Vega Abed	3	SWE	*a8*
Vega Abed	4	SWE	*a8*
Vega Abed	5	SWE	*a8, La*

[1] Accessions of 15 identical varieties from domestic gene bank (CZE) and their 75 SSPs written in italics were studied previously [3]. [2] Country of gene bank: CZE – Czech Republic, DEU – Germany, GBR – United Kingdom, HUN – Hungary, POL – Poland, SVK – Slovakia, SWE – Sweden, USA – United States.
